# A Multimodal Speech-Gesture Training Intervention for Patients With Schizophrenia and Its Neural Underpinnings – the Study Protocol of a Randomized Controlled Pilot Trial

**DOI:** 10.3389/fpsyt.2020.00110

**Published:** 2020-03-06

**Authors:** Lydia Riedl, Arne Nagels, Gebhard Sammer, Benjamin Straube

**Affiliations:** ^1^Translational Neuroimaging Lab, Department of Psychiatry and Psychotherapy, Philipps-University Marburg, Marburg, Germany; ^2^Department of English and Linguistics, Johannes-Gutenberg-University, Mainz, Germany; ^3^Department of Psychiatry and Psychotherapy, Justus-Liebig-University, Gießen, Germany

**Keywords:** schizophrenia, intervention, training, communication, speech, gesture, multimodal, fMRI

## Abstract

Dysfunctional social communication is one of the most stable characteristics in patients with schizophrenia that also affects quality of life. Interpreting abstract speech and integrating nonverbal modalities is particularly affected. Considering the impact of communication on social life but failure to treat communication dysfunctions with usual treatment, we will investigate the possibility to improve verbal and non-verbal communication in schizophrenia by applying a multimodal speech-gesture training (MSG training). Here we describe the newly developed MSG training program and the study design for the first clinical investigation. The intervention contains perceptive rating (match/mismatch of sentence and gesture) and memory tasks (n-back tasks), imitation and productive tasks (e.g., SG fluency—similar to verbal fluency where words are accompanied by gesture). In addition, we offer information about gesture as meta-learning element as well as homework for reasons of transfer to everyday life as part of every session. In the MSG training intervention, we offer eight sessions (60 min each) of training. The first pilot study is currently conducted as a single-center, randomized controlled trial of speech-gesture intervention versus wait-list control with a follow-up. Outcomes are measured through pre-post-fMRI and standardized psychological questionnaires comparing two subject groups (30 patients with schizophrenia and 30 healthy controls). Patients and healthy controls are randomized in two intervention groups (with 20 being in the wait-training group and 10 in the training-follow-up group). With our study design we will be able to demonstrate the beneficial effect of the MSG training intervention on behavioral and neural levels.

**Clinical Trial Registration:**

DRKS.de, identifier DRKS00015118.

## Introduction

Dysfunctional social communication is one of the most stable characteristics of patients suffering from schizophrenia that affects not only the personality of patients but also their quality of life. Schizophrenia is a chronic, often devastating disease with the following core symptoms according to DSM-5: delusions, hallucinations, disorganized speech, disorganized, or catatonic behavior and so called negative symptoms (e.g., the 3 A's: apathy, anhedonia, and alogia). These core symptoms are mostly treated by psychopharmacological medication. Unfortunately, medical treatment cannot help all patients similarly (1, 2). In particular the distinct social problems are part of the central issues in modern psychiatry, especially with regard to the bad outcome for life quality (3).

### Speech and Language Processing in Schizophrenia

Communication is the fundamental basis of social life. In schizophrenia, it is particularly affected: Two of the DSM-5 core symptoms of schizophrenia include speech disorders (disorganized speech and alogia—one of the negative symptoms). Patients suffer from speech and language aberrations on all linguistic levels. In acute phases, communication is extremely limited—some studies report parallels of schizophrenic speech and aphasia (4). Therefore, referring to the term “aphasia”, researchers call the productive speech of patients “schizophasia” (5). Further linguistic impairments are for instance an incoherent discourse, less referential cohesion and loosening of associations. These symptoms of disordered speech are evidence of formal thought disorder (6), which can also be a symptom of other psychiatric diseases, such as depression.

Not only the production of speech is impaired in schizophrenia, but also the general understanding and interpretation of the meaning of speech and language information is seriously affected. For instance, patients show difficulties in the understanding of figurative speech or abstract concepts being conveyed through metaphors, proverbs, humorous or ironic expressions, which they tend to misinterpret in a concrete way (7–11). Hence, this phenomenon is clinically termed “concretism” (12). In everyday life, metaphors are very frequently used to refer to abstract concepts such as feelings or events (e.g., “out of the blue”). Dysfunctional interpretation of metaphors thus have a huge impact on successful social interactions (13). By contrast, the understanding of concrete speech seems relatively unaffected in schizophrenia (14–16).

### Gesture Processing in Schizophrenia

As one integral feature of embodiment (17), gesture “serves as an outward manifestation of several interacting fundamental processes” (18) including speech perception (19, 20), memory (14, 21), and social functioning (22, 23). Therefore, dysfunction in the integration and interpretation of gesture information represents a core feature of disordered communication processes. Aberrations in gesture processing are found across all stages of schizophrenia, in production as well as in perception and interpretation. Patients use less and incoherent, mismatching gestures (24, 25). Furthermore, they tend to misinterpret gestures in a negative way (22, 26). Some authors therefore claim a general disruption of the integration of two modalities (auditory = speech and visual = gesture) in this disease (22, 26–29).

We can classify gestures—according to speech—in concrete gestures and figurative or abstract gestures: Concrete gestures, for example, are iconic gestures (e.g., literal gestures such as forming the shape of a dog's mouth with a hand while discussing a dog), abstract gestures are metaphorical hand and arm movements that accompany metaphors in speech (e.g., forming a cup with a hand while discussing a concept such as love). Moreover, similar to patients suffering from disordered speech, patients with schizophrenia also have trouble interpreting abstract meaning in gestures (30, 31). Strikingly, integration skills interact with symptomatology: Nagels and colleagues found worse integration skills, reflected in the evaluation of the semantic relationship between speech and gesture, in patients with severe symptoms compared to patients with mild symptom severity in formal thought disorders (32).

### Speech, Gesture and Its Neural Correlates

As mentioned above, decoding of abstract meaning is particularly challenging for patients with schizophrenia—on the level of auditive (speech) as well as in the visual (gesture) modality. Furthermore, the binding of information from multiple modalities (such as gesture and speech) is a complex unification process (33) which seems to be impaired in patients: Several studies have investigated the neural activity during the interpretation and integration of abstract, especially metaphoric, speech and gesture. According to these studies, neural activity correlates with the processing of abstract speech and the integration of gesture. Compared to healthy controls, patients with schizophrenia show abnormal activation, mostly in fronto-temporal regions, during the interpretation of metaphorical speech (10). During the integration of metaphoric gestures, patients with schizophrenia also show abnormal activation of and connectivity between frontal regions, especially in the inferior frontal gyrus (IFG) and middle and superior temporal areas (30, 31). Strikingly, aberrant neural activation, as well as reduced behavioral performance in communication tasks, has been found in medicated patients with chronic schizophrenia in most of the studies and hence seems to be stable despite of medication.

### Current Treatments for Patients With Schizophrenia

As outlined above, the exclusive medical treatment of schizophrenia is not effective for social-communicative functioning. Studies in the last 20 to 30 years have proved psychotherapeutic intervention to be an effective treatment of schizophrenia and at least support the medical treatment (3). Nowadays, several therapeutic programs exist which can efficiently complement the therapy of patients with schizophrenia. Along with psychotherapy (e.g., cognitive behavioral therapy), there exist occupational therapy (34, 35), physical therapy (36)/dance and movement therapy (37), and diverse art therapies (38) [e.g., art therapy—with inconsistent outcomes, however (39)—and music therapy (40–43)] for patients with schizophrenia.

So far, there have been few studies dealing with speech or communication therapy for patients with schizophrenia, although patients show problems in production and perception of speech and gesture integration (44). In a systematic review on speech language therapy in schizophrenia, Joyal and colleagues complain about speech language therapy not yet being a systematical part of a comprehensive intervention. They claim that speech and language deficits “might not always be the most preoccupying symptoms, in comparison with other symptoms such as hallucinations ” (45). Nevertheless, first studies are investigating the outcome of communication therapy in schizophrenia. There already exist some single subject studies showing positive effects of language and speech therapy on speech production in patients with symptoms such as alogia or delusional speech (46–49). Further studies have found positive effects for discourse production (score of intelligibility, appropriateness and elaboration of responses) (50), verbal fluency (51–53) and naming (54). On the other hand, some studies could neither find a benefit of speech language therapy for sentence understanding, repetition and naming (55), nor for verbal fluency (52, 56) and pragmatic non-verbal skills (57). However, except one of these studies, all previous studies focused on speech production, not on understanding language [for more information see the systematic review of Joyal and colleagues, mentioned above (45)]. According to the classic Wernicke-Lichtheim-Geschwind model, speech production is secondary to perception (58). Hence, in speech language therapy, for example in patients with aphasia after stroke where both modalities are affected, speech perception is treated first or at least parallel to the production of speech. Due to the fact that in schizophrenia understanding language is frequently affected, a training of speech perception and interpretation of meaning is appropriate.

Because some of the main communication problems of patients with schizophrenia happen on the pragmatic level, it is important to not only take into account isolated words or phrases, but whole sentences which create a context. It can be assumed that one of the communication problems in schizophrenia results from a lack of integrating words into larger units (59). The integration of words in context is strongly associated with working memory capacities, as Kintsch and van Dijk claim in their model of text comprehension and production (60). Hence, working memory and language processing seem to be thoroughly connected. This is also true for working memory and the integration of gesture (14, 21, 61). For that reason, not only sentence level but also specifically working memory for speech and gesture should be taken into account. For working memory (but not in the context of speech-gesture integration) there already exist some training approaches using n-back tasks (62–66).

Social communication is not only based on speech itself but also on the integration of nonverbal information such as gesture (67). Thus, a potential social communicative gesture training might help to develop, reactivate or promote communication resources in patients with schizophrenia. Nonverbal trainings are rare in the field of psychiatry. One study executed no training, but could show positive effects of transcranial direct current stimulation (tDCS) on gesture integration in a group of patients with schizophrenia spectrum disorder (68). Another study could show a benefit from single session transcranial magnetic stimulation (TMS) also for gesture production in patients with schizophrenia (69). An effect, which could be potentially increased and prolonged in combination with an adequate gesture training program. Despite the lack of previous studies about (perceptual) speech and gesture training for psychiatric patients, there is some evidence about effects of gesture training in other cognitive impairments such as aphasia (which is close to the speech symptoms in schizophrenia as one can see in the term “schizophasia” for speech and language problems in schizophrenia). In a systematic review from 2013, Rose and colleagues state that an isolated gesture training has no impact, but 50% of patients with aphasia are able to benefit from a combined speech-gesture-training. Some of the evaluated studies even showed generalization effects (70). Gesture cues (perceptual modality) also seem to have a positive outcome (71). The majority of the currently existing gesture trainings for patients with aphasia are based on the one word level and were shown to specifically improve naming performance. The influence of speech-gesture-training on the sentence level has scarcely been examined so far, but given the previous findings, it seems to be promising to implement such a speech-gesture-training for patients with schizophrenia.

Considering the potential impact of communication on social life and therefore life quality in general, it is particularly important to focus on the possibility of a holistic communication intervention.

### Development of a Multimodal Speech-Gesture Training

To solve the open questions regarding the efficiency of a specific MSG training in patients with schizophrenia, considering 1) natural communication—productive and perceptive, 2) working memory functions on sentence level and 3) integration of nonverbal communication, we developed a specific MSG training program, which will be evaluated with neural, behavioral and social outcome measures. Due to the possible impact dysfunctional communication has on life quality, we also evaluate transfer in everyday life social functioning.

For the first time, a specific MSG training has been tailored to the requirements of patients with schizophrenia. It is therefore important to assess whether the training is delivered as described in practice and whether the training is accepted by the patients. Given the potential difficulties in motivation and regularly attending sessions, we proposed offering eight sessions of high frequent intensive single speech-gesture-training to the participants. A high frequency of training sessions is also suggested by Joyal et al. for speech therapy in patients with schizophrenia (45). This number of sessions appears to be the minimum of sessions where participants can improve in such a novel training program (72).

The training was developed with focus on the main communication problems of persons diagnosed with schizophrenia. These include “concretism” (the inability to interpret abstract meanings considering context) and problems in nonverbal communication, especially in gesture accompanying abstract speech.

The setup of the MSG training sessions follows best practice in therapy intervention. Exercises are executed with increasing complexity (first perceptual, then productive and free productive tasks) and accompanied by an examiner. Items with concrete as well as abstract speech content are included in the exercises. To motivate the participants and to establish a trusting and respectful relationship between participant and examiner, the examiner introduces the training sessions with small talk and offers information about (nonverbal) communication. For reasons of transfer, the MSG training program includes homework which the participant is invited to do with a person he/she is in close contact.

### Estimation and Appropriateness of Outcome

We expect to achieve a range of positive behavioural (73), neural (74, 75) and social outcomes (76).

We measure behavioral outcomes through the perception of videos with speech and gesture during fMRI measurements. We further expect that initial difficulties in memorizing gesture and speech information can be reduced due to the MSG training so that behavioral performance in a speech gesture working memory task (see below) is more similar to healthy controls after training. In addition to the outcome measures, we explore improvement over time during the eight training sessions regarding speech-gesture matching performance [reaction times (RT) and percent correct], gesture-speech working memory performance (RTs and percent correct), SG fluency performance (number of correct items) and gesture imitation (experimenter evaluation of accuracy) in dependency from speech content (concrete/abstract).

We measure neural patterns through fMRI during the perception of videos with speech and gesture information using specific experiments with iconic (concrete) and metaphoric (abstract) gesture materials (15) as well as an n-back working-memory task. Furthermore, we correlate the behavioral results with neural activation patterns using fMRI techniques before and after the training. Similar to the behavioral outcomes, we expect the neural activity of the patients in relevant brain regions [specifically the left posterior temporal lobe and the left inferior frontal gyrus, cf. (15, 30, 75, 77)] to converge to the neural activity of healthy controls.

As communication is the basis for social interaction and hence for quality of life, transfer effects will be investigated through standardized psychological questionnaires and a specifically outlined questionnaire about nonverbal communication and social life.

However, we do not yet know which exercises and measurements might be most appropriate in terms of acceptability of completion and variability of outcome. We are going to provide descriptive statistics.

## Methods

### Aims and Objectives

This study aims to investigate the behavioral and neural effects of a new speech-gesture-training program for patients with schizophrenia.

The objectives are to:

1. Assess the acceptability of a MSG training in patients.
2. Assess the training effects in terms of n-back task performance for speech and gesture videos in dependency of speech content.
3. Assess the training effects on the neural correlates of speech-gesture integration and working memory in dependency of speech content.
4. Assess the behavioral outcomes of MSG training tasks in association to neural activation for each condition of stimuli (see stimuli section for more information).
5. Assess the effect of gesture training on scores of psychological questionnaires relating to social performance and quality of life.

### Setting and Enrollment

#### Setting

This is a single-center randomized controlled trial of intensive single speech-gesture training versus wait-list control with a follow-up being conducted at Philipps-University Marburg, Department of Psychiatry and Psychotherapy. In this institution, 30 participants are recruited per subject group (30 patients with schizophrenia and 30 healthy controls). Outcomes are measured through pre-post-fMRI and standardized psychological questionnaires comparing two subject groups (patients with schizophrenia and healthy controls) and two intervention groups (wait-training group and training-follow-up group).

#### Inclusion of Participants

In a telephone screening, interested persons are called by one of the researchers to make sure that both their status of physical health and capability to participate in an fMRI study meet our conditions. Inclusion and exclusion criteria are part of this screening as well as a questionnaire about the progress, the individual core symptoms of schizophrenia and medication in patients.

Subjects *are eligible* for study entry if they meet the following criteria:

- Aged between 18 and 60 years.
- Capacity to give informed consent.

Additional criteria, patients only:

- Diagnosis of schizophrenia after DCM-5 criteria.
- Relatively stable symptoms (no acute psychosis).

To evaluate the stadium and type of symptoms of the patients as well as for safety reasons, we additionally conduct a pre-scan interview with the patients including questions concerning the illness and medication as well as three standardized psychological questionnaires for schizophrenia [SAPS (78)/SANS (79) and PSP-Scale (80)].

Subjects *are not eligible* if any of the following criteria are present:

- No capacity to give informed consent.
- Risk of suicide necessitating hospitalization.
- Physical illnesses that interfere with the planned measurements.
- Medical contraindication against fMRI measurements.
- Pregnancy.
- Contraception per intrauterine contraceptive device.

Additional criterion for exclusion, control subjects only:

- Diagnosis of psychological diseases, especially schizophrenia.

If interested subjects meet the criteria, they meet with a member of the research team to go through the study information. Inclusion and exclusion criteria are confirmed along with the assessment of positive and negative symptoms of schizophrenia as well as gesture performance (for patients), social activity and quality of life (all participants). Participants are informed that they are free to withdraw at any time without giving reasons and without prejudicing any further treatment.

#### Intervention Groups and Randomization

In addition to the fMRI before and after the training programme, we conducted a further fMRI measurement after a period of waiting (no training = treatment as usual: TAU). For a variety of reasons, subjects in our study participate in both—the training programme and the treatment as usual. A first benefit from this approach is the comparability of both groups (perfect matching of the subjects). Secondly, we want to offer our training programme to all patients, which is possible woth our design where patients of the waiting list (TAU) group could also benefit from the programme.

**Figure 1:**
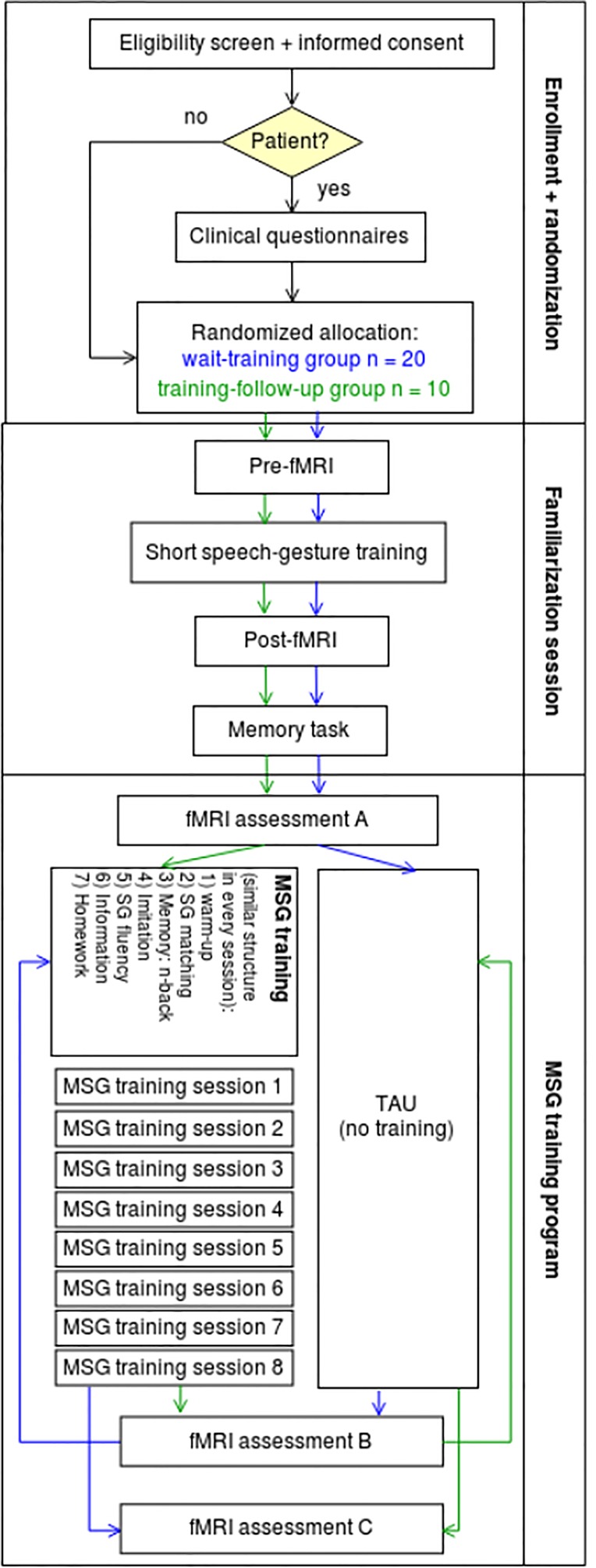
Flow diagram of our study to assess the neural activation and behavioral outcomes of a speech-gesture training in patients with schizophrenia and a healthy control group. All in all, 60 people will participate in this study: 20 patients with schizophrenia in the wait–training group, 10 patients in the training–following-up group, 20 healthy controls in the wait–training group, and 10 controls in the training–follow-up group.

For these reasons we do not divide patients and healthy controls in training and TAU group but in 1) wait-training group and 2) training-follow-up group. The first group initially participates in two pre-fMRI measurements with a waiting time in between (TAU time, assessment A and B) and after that participates in the training program with a post-fMRI measurement (assessment C); the second group starts by participating in the training with pre and post measurement (assessment A and B) and after a waiting time (TAU time) goes on with a follow-up measurement (assessment C, see **Figure 1**). To assess normal functioning on behavioral and neural level, healthy subjects are also involved as control group in our training procedure, so that we are able to compare the outcomes of the training.

With this design, altogether, we will be able to compare 30 patients and 30 control subjects pre-to-post (assessment A vs. C, see **Figure 1**) with an equal number of repetitions (3 measurements each) as well as data from MSG training program of all subjects. In order to obtain sufficient data on specific training effects from MSG training (compared to TAU effects, repetition or general time effects in the waiting list group), we decided to include at least 20 participants per subject group (20 patients and 20 control subjects) in the wait-training group (wait-list control: without training between first and second measurement) and explore possible long-term effects in a small group of ten subjects per subject group (training-follow-up group: without training between second and third measurement).

#### Criteria for Discontinuation

Participants are withdrawn from the intervention if the participants wish to be excluded for personal reasons, if participants become too unwell to continue, if the patients have a level of risk assessed by the clinical team to require hospitalization or if the team determines that the patient's current mental state, behavior or risk to self or others requires discontinuation of the intervention.

#### Sociodemographic Information

The 18 patients who finished the MSG training program and all associated assessments so far were diagnosed with schizophrenia (F20). Four of them were women, mean age was 34.9 years (SD = 10.9 years/range: 23–62 years). In everage they had attended school for 11.5 years (SD = 1.8 years).

All patients were recruited from and diagnosed by clinicians according to the Diagnostic and Statistical Manual of Mental Disorders, fifth edition (DSM-V) or the International Classification of Diseases, tenth edition (ICD-10). All patients were free of visual and auditory deficits, additional neurological and medical impairments as well as any cerebral abnormality, as assessed by a T1-weighted MRI. All patients reported that they were right handed and German was their primary language.

The study was approved by the Ethics Committee of the Philipps University Marburg and has been registered at the German Clinical Trials Register, DRKS (DRKS00015118, DRKS.de). All patients gave written informed consent and were paid 170 Euro for participation.

### Familiarization Session

To reduce dropout rates, we decided to undertake one familiarization session before we start with the actual training program and measurements. In this session, patients and control subjects are familiarized with the MRI environment and specific procedures of the MSG training procedure (specifically, with perceptive tasks and gesture imitation). For this reason, participants undergo two MRI measurements before (pre-fMRI) and after (post-fMRI) a short speech-gesture training of approximately 30 min. After the post-fMRI measurement, participants are asked to solve a memory task.

Before we start the measurement, the participants are informed about the setting of the study. A member of the research team will go through the study information and answer any questions. This familiarization procedure should reduce dropout rates due to problems with the scanning environment or other training related procedures (e.g., compliance regarding gesture production tasks).

#### fMRI Measurements

For the fMRI measurements, detailed manuals are provided to the researchers who conduct measurements. Furthermore, researchers are trained before they conduct parts of the study with participants. During fMRI measurements, two researchers are present. Protocols will be written for fMRI measurements.

FMRI measurements are conducted in this session before (pre-fMRI) and after (post-fMRI) a short speech-gesture training of 30 min.

#### Stimuli

In order to gain an advanced understanding in the engine of integration mechanisms and their neural correlates in patients with schizophrenia, participants undergo fMRI measurements watching videos on a screen. The videos were recorded with an actor expressing concrete or abstract sentences (for an explanation of concreteness see “gestures in schizophrenia” in the background section). The videos were standardized, extensively evaluated and had been successfully applied in a large number of fMRI (13–15, 81), EEG (82), tDCS (65, 83) and patient studies including patients with schizophrenia (28, 29). Altogether, we use two categories of videos in the familiarization session: videos containing.

1. concrete sentences without gesture (S_CON)
2. abstract sentences without gesture (S_ABS)

Videos from pre-fMRI measurement are repeated in post-fMRI measurement.

The videos’ sentences in the short speech-gesture training are repetitions from pre-fMRI measurement, but the videos this time are presented in two different modalities:

1. without gesture (auditive modality: S),
2. accompanied by gesture (bimodal: SG),

The videos were standardized, extensively evaluated and had been successfully applied in a study (84).

In the memory task after the post-fMRI measurement, sentences are presented to the participants in audio. The sentences are repetitions from the fMRI measurements and the short speech-gesture training.

We developed four different counterbalanced versions of stimuli presentation to avoid sequence effects.

#### fMRI Tasks

In the task during the pre- and post-fMRI measurement, participants are asked to decide whether the sentences presented to them were concrete or abstract (decision task). This allows us to compare the impact of concreteness and abstractness (due to concretism in schizophrenia) on behavioral outcome and neural activation.

#### fMRI Paradigm

The subjects are instructed to tap the fingers of their left hand on the buttons of a response box that is fixated at the subject's left leg. Subjects are asked to tap with their left forefinger for videos with concrete content and with their left middle finger for videos with abstract content.

Imaging data are collected with a 3 T whole body MRI system (SIEMENS MAGNETOM TrioTim syngo MR B17) equipped with a standard head coil. Structural image acquisition consists of 128 T1 weighted sagittal slices (slice thickness = 1.6 mm; FoV = 260 mm; TR = 3.15 s; TE = 1.37 s). To measure BOLD changes in brain activity during acquisition, T2* weighted gradient echo planar imaging (EPI) with 34 slices covering the whole brain will be used (voxel size = 3 x 3 x 4 mm; descending slice acquisition; slice thickness = 4.0 mm; TR = 1650 ms; TE = 25 ms; flip angle = 70°; FoV = 192 mm; GRAPPA = 2). Slices are adjusted after the anterior commissure posterior commissure (AC- PC) line. In the three measurements (A, B and C), 936 functional images are acquired during acquisition phase in the passive perception task and 666 in the memory task (n-back task). A gradient echo field map sequence is measured before the functional runs to get information for unwarping B0 distortions.

Data will be analyzed using standard procedures of Statistical Parametric Mapping (SPM12 (85), RRID: SCR_007037) implemented in MATLAB R2018b (MATLAB (86), RRID: SCR_001622). Unwarping and realignment, slice time correction, coregistration, segmentation and normalization to the standard space of the Montreal Neurological Institute brain (MNI-brain) as well as smoothing will be performed.

#### Short Speech-Gesture Training

Between the two fMRI measurements in the familiarization session, participants are trained with videos containing sentences with and without gesture. They are asked to attentively watch the videos. Some of the videos presented in bimodal condition (speech accompanied by gesture) are to be imitated by the participants.

#### Memory Task After Post-fMRI

After post-fMRI measurement, participants conduct a memory task, where audios from the sentences are presented, that also appeared in the fMRI measurement's and short speech-gesture training's videos. Participants are asked to decide whether they know the presented sentences from the short speech-gesture training and if yes, if the videos appeared with or without gesture and if the participants were asked to imitate the videos during the short training. That is, participants have four options to answer:

1. Sentence was not trained (unimodal control condition: S_C)
2. Sentence appeared in the training without gesture (unimodal perception: S_P)
3. Sentence appeared in the training with gesture (bimodal perception: SG_P)
4. Sentence with gesture was asked to be imitated in the training (bimodal imitation: SG_I)

### The MSG Training Program

#### Social and Quality of Life Questionnaires

To assess the effect of the gesture training on social functioning and quality of life, participants (patients and controls) are asked to complete psychological questionnaires about social life [SASS – Soziale Aktivität Selbstbeurteilungs-Skala (87): satisfying validity and reliability with cronbach alpha = 0.89 (88)] and quality of life [SWLS – Satisfaction with Life Scale (89): satisfying validity and reliability with cronbach alpha = 0.87 (81)] before and after the training program (assessment A/B and B/C, respectively). Furthermore, patients are asked about schizophrenia symptoms to assess positive and negative symptoms [(through SAPS (78)/SANS (79) and PSP-Scale (80)] as well as gesture performance [through the short version of TULIA—Test of Upper Limb Apraxia(82) and BAG—brief self-rating scale for the assessment of individual differences in gesture perception and production (83)] before they start with the training. The ratings in the questionnaires will later be correlated with the behavioral and neural outcome of the training to evaluate the effects on social life and quality of life.

#### fMRI Measurements (A, B, and C)

For the fMRI measurements, detailed manuals are provided to the researchers who conduct measurements. Furthermore, researchers are trained before they conduct parts of the study with participants. During fMRI measurements, two researchers are present. Protocols will be written for fMRI measurements.

#### Stimuli

In order to gain an advanced understanding in the engine of integration mechanisms and their neural correlates in patients with schizophrenia, participants undergo fMRI measurements watching videos on a screen^1^ before and after the actual training and waiting-list program (assessments A, B, and C). These videos differ from the ones presented in the familiarization session and from videos utilized during the MSG training procedure. The videos were recorded with an actor expressing concrete or abstract sentences (for an explanation of concreteness see “gestures in schizophrenia” in the background section). These sentences were recorded in three different modalities: accompanied by gesture (bimodal), without gesture (auditive modality) and gesture only/without speech (visual modality). The videos were standardized, extensively evaluated and had been successfully applied in a large number of fMRI (14–16, 84), EEG (85), tDCS (68, 86) and patient studies including patients with schizophrenia (30, 31). Altogether, we use six categories of videos in our study: videos containing.

1. concrete sentences accompanied by gesture (SG_CON)
2. abstract sentences accompanied by gesture (SG_ABS)
3. concrete sentences without gesture (S_CON)
4. abstract sentences without gesture (S_ABS)
5. concrete gesture without speech^2^ (G_CON)
6. abstract gesture without speech (G_ABS)

Hence, there are three conditions of modality (bimodal, visual modality, auditive modality) and two conditions of concreteness (concrete and abstract) in our stimuli.

Videos of the n-back task comprise sentences with deictic content, containing pointing gestures. Again, these videos were recorded in three different modalities:

1. accompanied by deictic gesture (bimodal: SG),
2. without gesture (auditive modality: S),
3. gesture only/without speech (visual modality: G).

These videos were standardized, extensively evaluated and had been successfully applied in a study (90).

To investigate repetition effects, the stimuli in the three fMRI measurements (A, B and C) are balanced: 50% of the videos are repeated at each assessment and 50% of the videos are completely new.

We developed eight different counterbalanced versions of stimuli presentation to avoid sequence effects.

#### Tasks

In a first task, the videos are presented to the participants who simply should confirm that they attentively watched (passive task). This allows us to compare the impact of concreteness and abstractness (due to concretism in schizophrenia) and different modalities [auditive vs. visual modality and the integration of both: cf. (15, 31)] on behavioral outcome and neural activation. In a second task, participants conduct an n-back task (one back and two back), where again different videos (speech, gesture and speech-gesture videos) are presented, but some of them are repeated directly (one back) or indirectly/with one new video in between [two back: see (61) for a review about a comparable approach].

#### fMRI Paradigm

The subjects are instructed to tap the fingers of their left hand on the buttons of a response box that is fixated at the subject's left leg. In the first task (passive task), subjects are asked to tap with their left forefinger to confirm that they watched the video (and to ensure they stay awake). In the second task (n-back task), participants are asked to tap with their left forefinger or their left middle finger, depending on their answer if the presented video seems to be a new or a repeated video.

Imaging data are collected with a 3 T whole body MRI system (SIEMENS MAGNETOM TrioTim syngo MR B17) equipped with a standard head coil. Structural image acquisition consists of 128 T1 weighted sagittal slices (slice thickness = 1.6 mm; FoV = 260 mm; TR = 3.15 s; TE = 1.37 s). To measure BOLD changes in brain activity during acquisition, T2* weighted gradient echo planar imaging (EPI) with 34 slices covering the whole brain will be used (voxel size = 3 x 3 x 4 mm; descending slice acquisition; slice thickness = 4.0 mm; TR = 1650 ms; TE = 25 ms; flip angle = 70°; FoV = 192 mm; GRAPPA = 2). Slices are adjusted after the anterior commissure posterior commissure (AC- PC) line. In the three measurements (A, B, and C), 936 functional images are acquired during acquisition phase in the passive perception task and 666 in the memory task (n-back task). A gradient echo field map sequence is measured before the functional runs to get information for unwarping B_0_ distortions.

Data will be analyzed using standard procedures of Statistical Parametric Mapping (SPM12 (91), RRID: SCR_007037) implemented in MATLAB R2018b (MATLAB (92), RRID: SCR_001622). Unwarping and realignment, slice time correction, coregistration, segmentation, and normalization to the standard space of the Montreal Neurological Institute brain (MNI-brain) as well as smoothing will be performed.

#### Multimodal Speech-Gesture Training: MSG Training

##### Examiners

The MSG training has been described in detailed manuals developed for the purposes of this study and are provided to the researchers who conduct the trainings. Speech language therapists, linguists, psychologists and medical PhD students with their focus on psychiatric disorders are involved in the training and are trained before they conduct parts of the study (e.g., the training) with participants. Examiners do not alternate between participants to ensure steadiness. During MSG training sessions, at least one examiner is present. Protocols are written during the trainings. Examiners were unaware about our specific neural and behavioral hypothesis regarding the MSG training effects on the different measures.

##### Setting

Trainings take place in an individual setting for reasons of acceptability by patients, taking into account that exercises including gesture performance are extraordinary and therefore possibly hard to execute when other participants are present. We offer eight sessions (60 min each) of training for reasons of a high drop out rate in trainings with more sessions in previous studies (93). These training sessions are offered with a high frequency (three to five trainings per week) as interventions in communication skills seem to be more efficient in high frequency interventions with short durations than in low frequency interventions with long durations in patients with schizophrenia (45). A higher frequency is not possible in our design because the training includes homework for reasons of transfer, offering the opportunity to do the homework exercises between two sessions.

##### MSG Training Procedure

Every session has a similar sequence of exercises, following best practice in therapy intervention. A session begins with small talk to establish a respectful relationship between participant and examiner and with a discussion about the homework prepared for the current session. Thereafter, participants should execute four exercises with increasing complexity (first two perceptual tasks, then a productive (imitation/mime) and a free productive (SG fluency) task. As in the fMRI measurements, video material is used where an actor produces sentences with a concrete or abstract meaning and accompanies these sentences with gesture. The videos differ from the stimuli in the fMRI measurements.

In the first perceptual task, the videos are presented through a laptop screen. Participants are supposed to rate the matching of gesture and speech content [see (68, 86)]. Considering the connection between speech/gesture perception and working memory (14, 21, 61), the second perceptual task is a speech-gesture-n-back task (one back, two back and three back with speech, gesture and speech + gesture videos).

In the first productive task, participants are asked to imitate the information in the videos presented to them or mime a given word/concept. In the second productive task, the SG fluency task, a semantic field is given to the participants (e.g., tools). In the style of verbal fluency tasks (94), participants have one minute to produce as many words as they can in this field of words and accompany these words by suitable gesture.

Considering the high impact of motivation on the positive outcomes of an intervention (95), participants are provided with some interesting background information about gesture and how it is related to language and communication (“what it is good for”) that is supposed to motivate them to attend to and talk about gestures in everyday life situations.

Handouts summarizing the content of this information and an explanation of the new homework are given to participants at the end of each session which hopefully allows them to transfer the training effects to their daily life routine.

#### Treatment as Usual: TAU

In addition to the fMRI before and after the training programme, we conducted a further fMRI measurement after a period of waiting (no training = treatment as usual: TAU). This will allow us to compare the outcomes from the training to a period of no training. This approach is similar to a training-TAU-design with two groups (one undergoing the training and a control group undergoing the treatment as usual, which means no training in our case).

### Outcome Measures and End Points

Our interest is the behavioral outcome of an MSG training on communication, social skills and quality of life as well as its neural correlates in brain regions.

Proposed behavioral outcomes are:

Behavioral data:
  1. Behavioral outcomes (n-back task performance: accuracy and reaction time for each condition) in interaction with treatment group (wait-training group vs. training-follow-up group) and time point (assessment A vs. B and B vs. C)
  2. Behavioral outcomes (n-back task performance: accuracy and reaction time for each condition) in interaction with subject group (patients vs. control group) and time point (assessment A vs. C)
  3. Behavioral improvement during training (speech-gesture matching and working memory performance: reaction time and percent correct, SG fluency performance: number of correct items and gesture imitation: experimenter evaluation of accuracy) as interaction with group (patients vs. control group) and time point (training session one to eight)
Neural data:
  1. Neural activation (whole brain and specific regions of interest [ROI]: the left inferior frontal gyrus [IFG]) during the passive viewing and the n-back task in interaction with video condition, treatment group (wait-training group vs. training-follow-up group) and time point (Assessment A vs. B and B vs. C)
  2. Neural activation (whole brain and specific ROIs: STS and left IFG) during the passive viewing and the n-back task in interaction with video condition, subject group (patients vs. control group) and time point (Assessment A vs. C)
Social data (self-report):
  1. Social functioning as measured by SASS – Soziale Aktivität Selbstbeurteilungs-Skala(87) in interaction with time point (*t_3_* vs. *t_5_*) and group (patients vs. control group)
  2. Quality of life as measured by SWLS – Satisfaction with Life Scale(89) in interaction with time point (*t_3_* vs. *t_5_*) and group (patients vs. control group)
  3. Communication skills as measured by a questionnaire for relatives of the patients (created for this study by our research team) in correlation with neural and behavioral measures of MSG related improvements (e.g., *t_3_* vs. *t_5_*)

In addition to that, we are planning to correlate the neural and behavioral outcomes as well as neural and social and behavioral and social outcomes.

**Figure 2:**
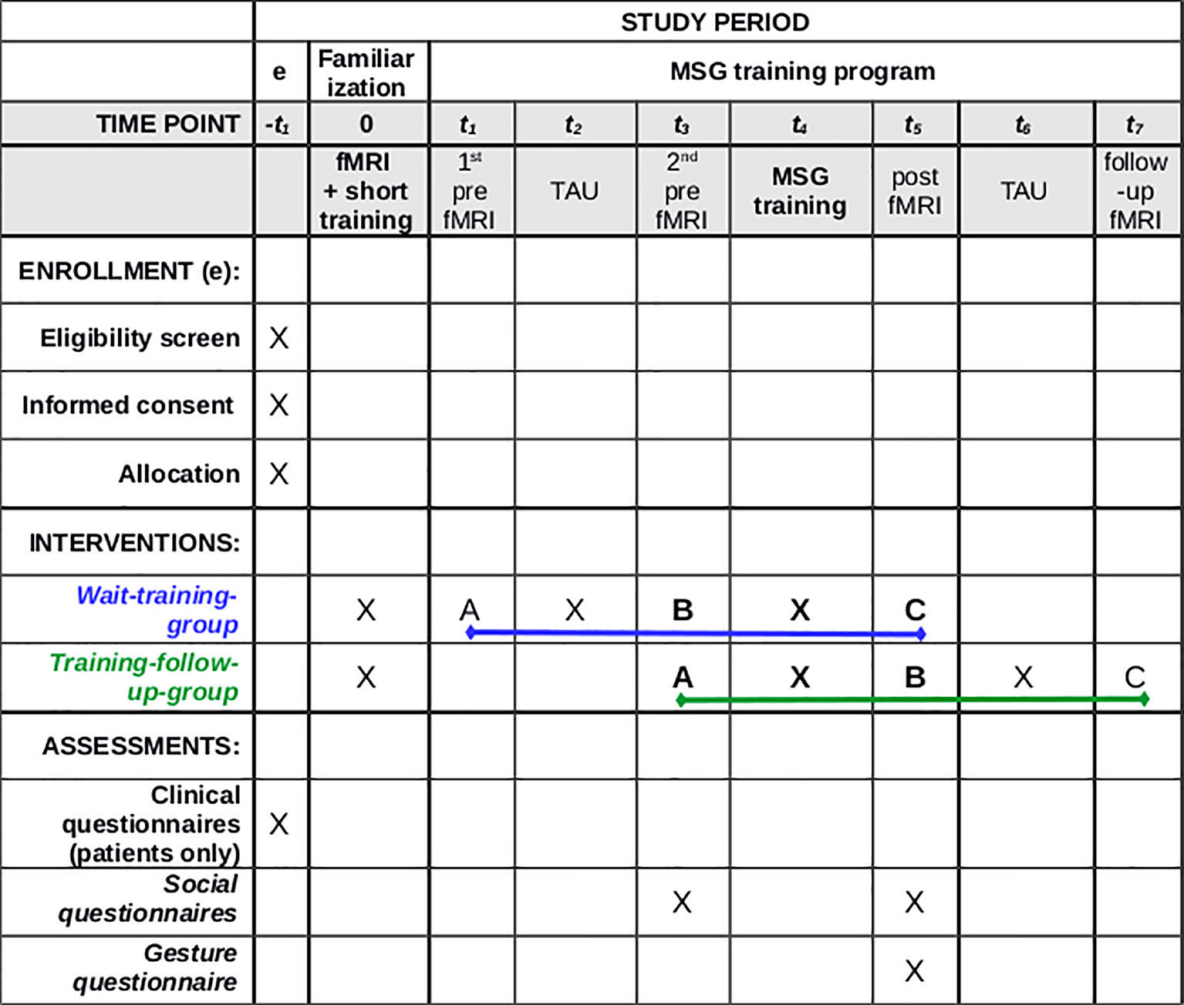
Standard protocol of our study. (A) Assessment A (first fMRI measurement); (B) Assessment B (second fMRI measurement); (C) Assessment C (third fMRI measurement); TAU, treatment as usual (waiting time without training intervention). Clinical questionnaires—SAPS, Scale for Assessment of Positive Symptoms; SANS, Scale for Assessment of Negative Symptoms; PSPS, Personal and Social Performance Scale; TULIA, Test of Upper Limb Apraxia; Social Questionnaires: SASS, Soziale Aktivität Selbstbeurteilungs-Skala; SWLS, Satisfaction with Life Scale; Gesture Questionnaire: questionnaire for relatives of the patients (created for this study by our research team).

For a summary/overview of the study see **Figure 2**.

### Trial Status

The study started first fMRI measurements (assessment A) on 21^st^ December 2017 and after treatment as usual and the second fMRI measurement (assessment B), the training started on 20^th^ January 2018 with participants of the wait-training group. So far (end of March 2019), 18 patients and three healthy controls have been measured and completed the MSG training. With a sample of this size we cannot calculate any group differences yet. This will be done as soon as the samples are complete. After the first participants had completed measurements and training, we evaluated our design and the training make-up. By majority, participants were satisfied with the training and so far, we had only one dropout. Satisfaction can be shown through data from our standardized post training questionnaire, where 13 participants answered that the training was very satisfying, five answered that it was satisfying, no one of the patients who finished the training was unsatisfied with the training. Concerning transfer into everyday life social functioning, the training was rated as very relevant from five patients and as relevant from another five patients. Four patients where not sure, three patients rated the training as less relevant and one patient rated the training as not relevant at all. Regarding the ratings from the patients and considering the high rate of dropouts in other training studies involving patients with schizophrenia (93), we evaluated our design and training as feasible for this group of patients.

## Discussion

### Current Study

As one aspect of embodied communication, (17) gesture is an integral feature of interpersonaldirect nonverbal communication that serves as an outward manifestation of several interacting fundamental processes. The binding of information from gesture and information from speech is a complex unification process (33) which seems to be impaired in schizophrenia. Especially metaphorical gesture processing seems to be disordered (30). Given the fact that in everyday life, metaphors are very frequently used to refer to abstract concepts, (13) a dysfunctional processing of abstract gestures affects integral elements of social skills which may lead to social isolation and therewith to a reduced life quality. These social-communicative deficits are accompanied by aberrations in neural activation (12, 30). Especially hypofrontality (96) and fronto-temporal disconnection (97) are reported in schizophrenia.

Unfortunately, these deficits in interpersonal communication cannot be significantly improved by medication, which might lead to persistent social isolation. Despite this fact, until now, scarcely any therapies can be found which consider specifically communicative and pragmatic skills for those who suffer from schizophrenia (98). Here we provide a first framework, training-procedural description and information about the current study design to solve the open questions regarding the efficiency of a specific MSG training in patients with schizophrenia. In our program, we consider 1) natural communication – productive and perceptive, 2) working memory functions on sentence level and 3) integration of nonverbal communication as well as the important transfer into everyday life social functioning. Furthermore, our newly developed MSG training program will be evaluated with neural, behavioral and social outcome measures, to directly relate dysfunctional neural mechanisms to the potential improvement expected in the intervention groups.

The present study offers an intensive MSG training to patients with schizophrenia and a healthy control group and correlates the behavioral outcomes from this training with neural activation before and after the training as well as with social skills and quality of life. The randomized controlled trial with waiting list and follow-up measurement enables us to demonstrate not only the effect of the gesture training on behavior, self-reports and neural correlates but also to explore long-term effects and potential predictors of treatment outcome (44, 98). While it remains to be seen whether changes can be detected within our proposed measures, the intervention seems at least to be profitable to the patients, who have completed the measurements and trainings so far (see section 2.6 *Trial Status*).

### Previous Studies

Previous evidence on training of communicative and pragmatic skills in schizophrenia suggest that it may be an acceptable and tolerable intervention to conduct an intensive MSG training (45). For example, some single subjects studies have shown positive effects of language and speech therapy on speech production in patients with symptoms such as alogia or delusional speech (46–49). Further studies have found positive effects for discourse production (50), verbal fluency (51–53) and naming (54) However, these studies focused on speech production, not on understanding language. According to the Wernicke-Lichtheim-Geschwind model, speech production is secondary to perception (58). Due to the fact that in schizophrenia understanding language is frequently affected, a training of speech perception and interpretation of meaning is appropriate. A multimodal communication training which also addresses gesture and speech perception may offer the opportunity to complement the currently recommended treatments and enable the patients to broaden their experiences. However, there is no evidence on the feasibility, effectiveness, behavioral, social or neural outcomes of such a training.

Since there is rarely evidence of how to design an effective multimodal communication training intervention for patients with schizophrenia, the design of our novel MSG training is mainly based on findings from aphasia treatment and speech therapy in the context of other cognitive deficits (e.g., on findings from the gesture training in aphasia review from Rose and colleagues (79).

As previously shown, interventions such as psychotherapy can change functional neural processes in clinical populations (99) including psychosis (100) For example, it has been shown that cognitive behaviour therapy for psychosis decreased activation of the inferior frontal, insula, thalamus, putamen and occipital areas to fearful and angry expressions at treatment follow-up compared with baseline. In line with this study, fMRI will be used to demonstrate training effects in two paradigms.

### Advantages

Highlighting the advantages of our novel MSG training intervention, in contrast to regular interventions, coupling of social-communicative skills along with motoric features are directly trained in our program. Beyond that, extra-linguistic and pragmatic information found in meaningful hand and arm movement interpretation is addressed. The playful character of the exercises simultaneously trains neurophysiological capacities and may result in a generalisation of possible training effects. Concerning the data that we collect, one of the current study's strengths is the brain imaging techniques that allow us to investigate the neural plasticity effects of the MSG training on social-cognitive abilities in patients with schizophrenia. Furthermore, we focus on cognitive aspects of gesture to facilitate social-cognitive functioning in everyday life. Due to the possible impact dysfunctional communication has on life quality, transfer in everyday life social functioning is one of our main objectives.

### Limitations

Limitations might be that we cannot eliminate effects of medication (changes in antipsychotic treatment) or possible (co-)influence of other therapeutic interventions which may influence the study results. Furthermore, some of the behavioral data that we collect are based on subjective self-given information from the patients and/or their relatives. In addition, the study is rather comprehensive and long-lasting, asking patients not only to train but also to participate in neural imaging assessments. This complex study design might result in a high number of dropouts from patients with severe symptoms. So far, at least from the patients who were willing to participate in our study, only one out of 18 patients stopped to participate prematurely.

### Future Studies

Approaches using noninvasive brain stimulation techniques such as transcranial direct current stimulation (tDCS) (68) and transcranial magnetic stimulation (TMS) (66), already showed positive effects on gesture integration and gesture production in patients with schizophrenia, respectively. This is an effect, which could be potentially increased and prolonged in combination with an adequate multimodal speech-gesture training intervention.

With the help of functional Magnetic Resonance Imaging (fMRI) and behavioral measures, further implications for future therapy could be investigated, in order to improve treatment methods for patients with schizophrenia.

## Conclusions

In our study, a specific MSG training is tailored to patients with schizophrenia for the first time. Given the previous findings about the compelling impact of gesture on communication and the influence of trainings on gesture and other pragmatic skills, it seems to be promising to implement such a training on patients suffering from schizophrenia. Considering the serious communicative and social problems of patients and the insufficient medical treatment of communicative and social disabilities, the invention of a communicative training program is sorely needed.

In the described study design we extend and combine different forms of therapeutic knowledge, as motoric and linguistic as well as extra-linguistic skills are trained. Moreover, imaging techniques are used to find neural evidence for improvement and changes on a neural level.

## Ethics Statement

This study was carried out in accordance with the recommendations of the local ethics committee (Philipps-University Marburg, Department of Medicine, Deanery/Ethics Committee, Reference: R1, Study 01/17) on 28th February 2017 with written informed consent from all subjects. All subjects gave written informed consent in accordance with the Declaration of Helsinki. The protocol was approved by the local ethics committee. All information collected is kept confidential, stored securely and archived in accordance with the research governance policy of the university. Participant anonymity is retained by allocating a unique identification number for the trial and any identifiable information stored separately from this.

## Author Contributions

LR, BS, and AN conceived the study and participated in its design. LR participated in the coordination of the study, drafted the manuscript and was the major contributor in writing. GS participated in conceiving the study. All authors read and approved the final manuscript.

## Funding

This article presents independent research funded by the von behring|röntgen|foundation (project number: ***64-0001***). The first author is funded by a scholarship of the Heinrich Böll Foundation. The views expressed are those of the authors and not necessarily those of the von behring|röntgen|foundation or Heinrich Böll Foundation.

## Conflict of Interest

The authors declare that the research was conducted in the absence of any commercial or financial relationships that could be construed as a potential conflict of interest.

## Footnotes

1 In MRI an additional mirror has to be used for presentation.

2 Due to the fact that gestures can only be classified considering the speech they accompany, we classified gesture without speech according the associated sentence in the other modality conditions.

## Supplementary Material

Supplementary Table 1 SPIRIT 2013 Checklist: Recommended items to address in a clinical trial protocol and related documents.

## Abbreviations

a, allocation; A, Assessment; e, enrollment; fMRI, functional magnetic resonance imaging; MSG training, multimodal speech-gesture training; PSPS, Personal and Social Performance Scale; SANS, Scale for Assessment of Negative Symptoms; SASS, Soziale Aktivität Selbstbeurteilungs-Skala; SAPS, Scale for Assessment of Positive Symptoms; SG fluency, speech-gesture fluency; SWLS, Satisfaction with Life Scale; TAU, treatment as usual; tDCS, transcranial direct current stimulation; TULIA, Test of Upper Limb Apraxia.
